# Factors associated with undertriage in patients classified by the need to visit a hospital by telephone triage: a retrospective cohort study

**DOI:** 10.1186/s12873-021-00552-x

**Published:** 2021-12-15

**Authors:** Ryota Inokuchi, Xueying Jin, Masao Iwagami, Toshikazu Abe, Masatoshi Ishikawa, Nanako Tamiya

**Affiliations:** 1 Department of Health Services Research, Faculty of Medicine, University of Tsukuba, 1-1-1 Tenno-dai, Tsukuba, Ibaraki, 305-8575 Japan; 2 Health Services Research and Development Center, University of Tsukuba, 1-1-1 Tenno-dai, Tsukuba, Ibaraki, 305-8575 Japan

**Keywords:** Triage, Undertriage, After-hours house call, Out-of-hours services, Emergency department

## Abstract

**Background:**

Prehospital telephone triage stratifies patients into five categories, “need immediate hospital visit by ambulance,” “need to visit a hospital within 1 hour,” “need to visit a hospital within 6 hours,” “need to visit a hospital within 24 hours,” and “do not need a hospital visit” in Japan. However, studies on whether present and past histories cause undertriage are limited in patients triaged as need an early hospital visit. We investigated factors associated with undertriage by comparing patient assessed to be appropriately triaged with those assessed undertriaged.

**Methods:**

We included all patients classified by telephone triage as need to visit a hospital within 1 h and 6 h who used a single after-hours house call (AHHC) medical service in Tokyo, Japan, between November 1, 2019, and November 31, 2020. After home consultation, AHHC doctors classified patients as grade 1 (treatable with over-the-counter medications), 2 (requires hospital or clinic visit), or 3 (requires ambulance transportation). Patients classified as grade 2 and 3 were defined as appropriately triaged and undertriaged, respectively.

**Results:**

We identified 10,742 eligible patients triaged as need to visit a hospital within 1 h and 6 h, including 10,479 (97.6%) appropriately triaged and 263 (2.4%) undertriaged patients. Multivariable logistic regression analyses revealed patients aged 16–64, 65–74, and ≥ 75 years (adjusted odds ratio [OR], 2.40 [95% confidence interval {CI} 1.71–3.36], 8.57 [95% CI 4.83–15.2], and 14.9 [95% CI 9.65–23.0], respectively; reference patients aged < 15 years); those with diabetes mellitus (2.31 [95% CI 1.25–4.26]); those with dementia (2.32 [95% CI 1.05–5.10]); and those with a history of cerebral infarction (1.98 [95% CI 1.01–3.87]) as more likely to be undertriaged.

**Conclusions:**

We found that older adults and patients with diabetes mellitus, dementia, or a history of cerebral infarction were at risk of undertriage in patients triaged as need to visit a hospital within 1 h and 6 h, but further studies are needed to validate these findings.

**Supplementary Information:**

The online version contains supplementary material available at 10.1186/s12873-021-00552-x.

## Introduction

### Background

Prehospital telephone triage systems identify the appropriate care required for patients and determine their need for a hospital visit or an immediate hospital visit by ambulance [1]. Telephone triage plays a pivotal role in managing hospital workload and patient flow [2], and many countries have adopted telephone triage protocols [2–4]. Similarly in Japan, the Fire and Disaster Management Agency developed telephone triage protocols and established telephone consultation centers in several large cities, including Tokyo, to provide telephone triage to patients [5].

Recently, to reduce overcrowding in emergency departments, many countries have launched after-hours house call (AHHC) medical services or out-of-hours services [6] because delays in care for patients increase the risk of serious complications [7].

Similarly, a private AHHC service was established in Tokyo, Japan. This service provides consultations through telephone triages using developed telephone triage protocols. These protocols classify patients into five categories: “need immediate hospital visit by ambulance (red),” “need to visit a hospital within 1 hour (orange),” “need to visit a hospital within 6 hours (yellow),” “need to visit a hospital within 24 hours (green),” and “do not need a hospital visit (white).” The AHHC or out-of-hours medical services target patients triaged as “need to visit a hospital within 1 hour (orange)” and “need to visit a hospital within 6 hours (yellow).”

Previous telephone triage studies on AHHC medical services have explored the safety, efficiency, or quality of records documented by the nurse or doctor during telephone triage [8–11]. However, to the best of our knowledge, no study has assessed whether patients’ present and past histories cause undertriage, especially in patients who are classified under the “need to visit a hospital within 1 hour (orange)” and “need to visit a hospital within 6 hours (yellow)” category by telephone triage.

We aimed to investigate risk factors for undertriaging by comparing patients who are classified as need an early hospital visit from the appropriately triaged and undertriaged groups using records from a single AHHC medical service.

## Methods

### Study design

This study had a retrospective design and used anonymized data from medical records of patients who used the AHHC medical service from November 1, 2019, to November 31, 2020. The study design was reviewed and approved by the Research Ethics Committee of the University of Tsukuba (approval number: 1527).

### Telephone triage system in Tokyo, Japan

A well-used telephone triage protocol developed by the Fire and Disaster Management Agency functions as follows: when a patient calls an emergency telephone consultation service, an operator classifies the patient into one of the five categories (red, orange, yellow, green, or white) based on a telephone triage protocol [5, 12, 13]. The protocols are divided into two categories: one for patients aged < 16 years and the other for those aged ≥16 years.

### AHHC medical service

We used a private AHHC medical service in Tokyo, established by Fast Doctor Ltd. (Shinjuku, Tokyo, Japan) in 2016, which sends doctors directly to the residence of patients who need a hospital visit. The company operates 7 days a week outside of regular hospital visiting hours (18:00 to 06:00 on weekdays and Saturdays and 24 h on Sundays and holidays).

When a patient calls the AHHC service, operators (trained telephone triage nurses) perform the telephone triage using the telephone triage protocol. The operators determine the following: whether the patient is to remain at home (white), whether an AHHC doctor’s visit to the patient’s residence is needed (orange and yellow), or whether they need an ambulance (red). The operators call an ambulance for patients triaged as red and opt to provide the patient with information about nearby clinics or a primary hospital for patients triaged as green. The trained telephone triage nurses can consult a doctor beside the AHHC medical service call center.

After the AHHC medical service doctors conduct home visits for patients, these doctors classify patients into three categories: grade 1 (can be treated using over-the-counter medications), grade 2 (require a hospital or clinic visit), or grade 3 (require ambulance transportation) [14–16].

The doctors affiliated to the AHHC medical service are specialized attending doctors working at a university hospital and aged approximately 35 years. The telephone triage nurses are trained to use the telephone triage protocol in the AHHC medical service.

**Fig. 1:**
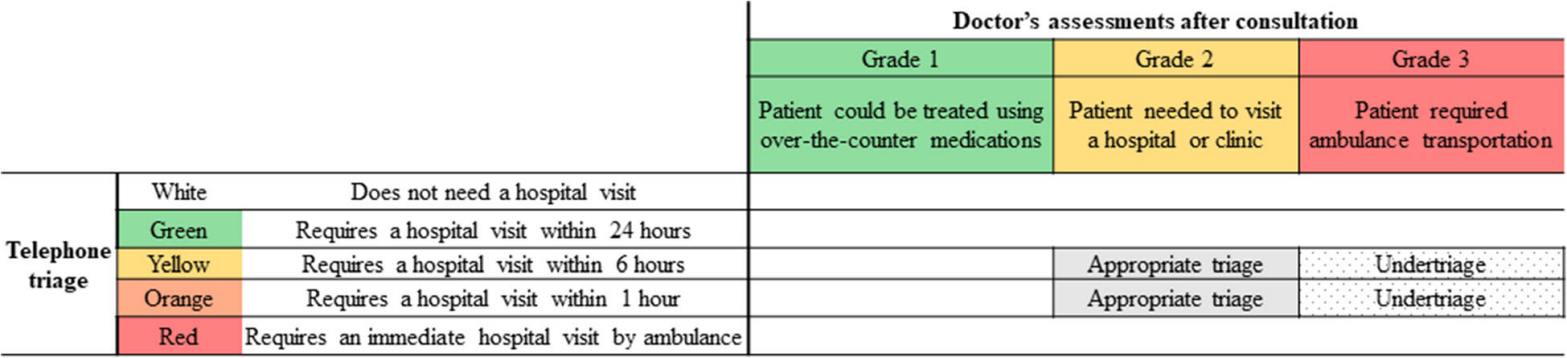
Flowchart of the selection of study patients

Figure 1 shows a correspondence table describing telephone triage and the grades assessed by AHHC doctors. Patients who were initially classified as orange and yellow and subsequently classified as grade 2 by AHHC doctors formed the appropriately triaged group. Patients initially classified as orange and yellow and subsequently as grade 3 by AHHC doctors formed the undertriaged group.

### Study participants

We included all patients who had used the AHHC medical services between November 1, 2019, and November 31, 2020, and excluded those with missing records of age, sex, telephone triage categories, or doctor grade assessments after consultations.

### Data source

The study used anonymized data from medical records of patients using the AHHC medical service (Fast Doctor Ltd.).

Data on the following variables of patients were extracted from medical records: sex, age, comorbidities (hypertension, diabetes mellitus, hyperlipidemia, gout, chronic lung disease, heart failure, liver disease, cerebral infarction, cancer, and dementia), telephone triage categories, protocols, doctor assessments after consultations, vital signs, and time from patients’ phone call to doctors’ consultations.

### Statistical analyses

First, we compared baseline characteristics between the appropriately triaged and undertriaged groups. Pearson’s chi-square test or Fisher’s exact test was performed to compare categorical variables (age categories [0–15, 16–64, 65–74, and > 75] years, sex, comorbidities, telephone triage categories, protocols, and doctor assessments), and Student’s *t*-test or the Wilcoxon-Mann-Whitney test was performed to compare continuous variables (age, vital signs, and time from telephone triage to patient consultation), as appropriate. Second, we conducted a multivariable logistic regression analysis to identify factors associated with undertriage. Third, we conducted sensitivity analyses to exclude patients aged 0–15 years because the protocol used for these patients is different from that used for patients aged > 15 years. All statistical analyses were performed using JMP 16.0 statistical software (SAS Institute, Cary, NC, USA). The significance threshold was set at *P* of < 0.05.

## Results

### Proportion of undertriage

**Fig. 2:**
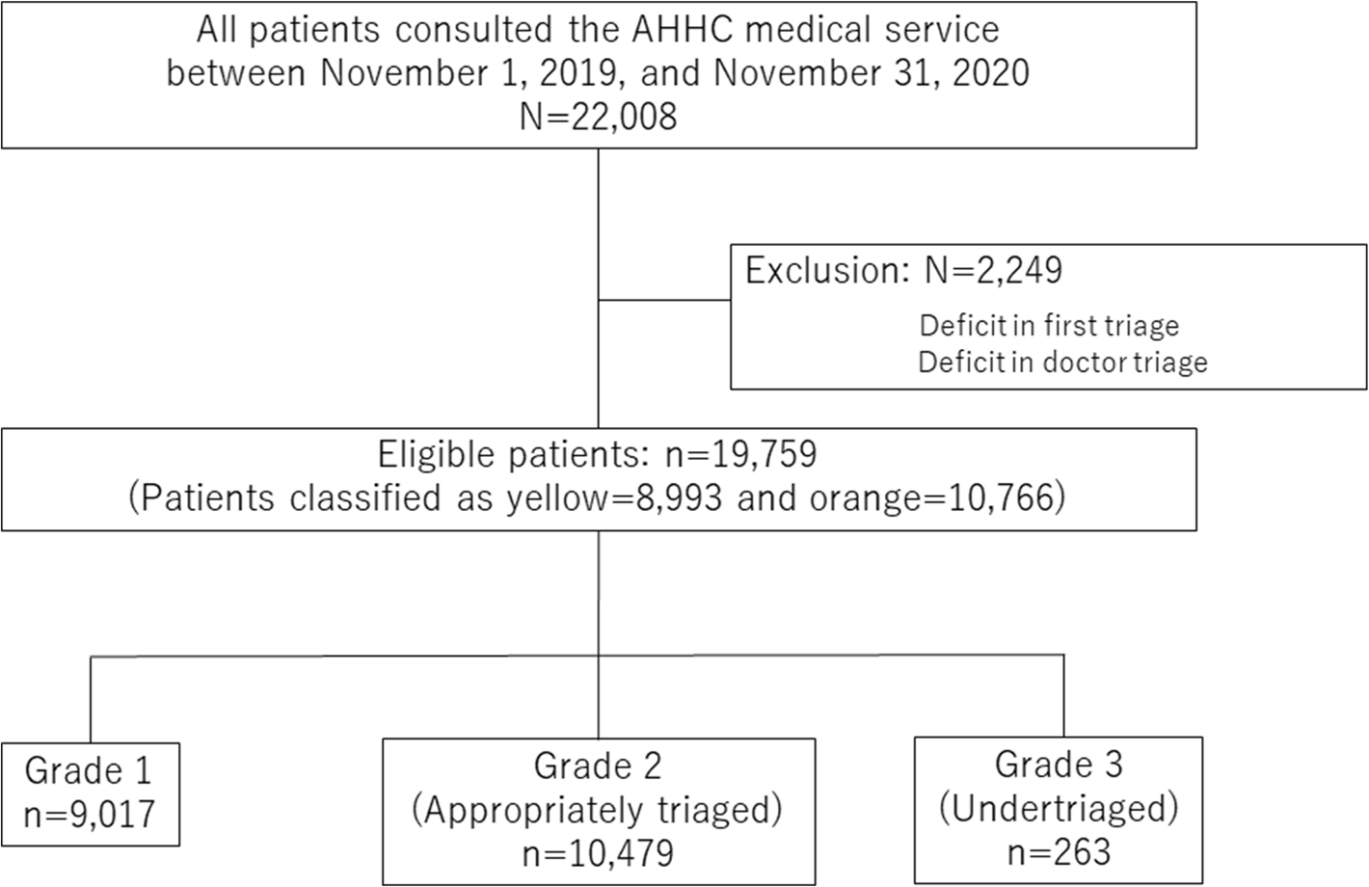
Definitions of appropriate triage and undertriage. When a patient calls the AHHC medical service, an operator classifies the patient into one of the five categories (red, orange, yellow, green, or white; at the left of the table). Doctors from the AHHC medical service visited patients who were classified as red, orange, and yellow and assessed them as grades 1, 2, or 3 after home visit and consultation (at the top of the table). Patients who were initially classified as orange and yellow and subsequently classified as grade 2 by AHHC doctors formed the appropriately triaged group. Patients who were initially classified as orange and yellow and subsequently as grade 3 by AHHC doctors formed the undertriaged group. AHHC, after-hours house call

A total of 22,008 patients consulted the AHHC medical service between November 1, 2019, and November 31, 2020. We excluded 2249 patients owing to the lack of data on telephone triage, leaving a total of 19,759 patients classified as yellow or orange. After AHHC doctor consultation, 9017 (45.6%), 10,479 (53.0%), and 263 (1.3%) were classified as grade 1, grade 2 (appropriately triaged), and grade 3 (undertriaged), respectively (Fig. 2 and Supplementary File 1).

### Comparing patient characteristics between the appropriately triaged and undertriaged groups

Among the 19,759 patients, we compared patients who were classified in the appropriately triaged (10,479 patients) and undertriaged (263 patients) groups. The patient characteristics are shown in Table 1.

**Table 1:** Bivariate analysis of appropriately triaged and undertriaged patients

	Appropriate triage*N* = 10,479	Undertriage*N* = 263	*p* value
Age, years			
Median (IQR)	21 (4–37)	46 (26–78)	< 0.001
Category, n (%)			< 0.001
0–15	4960 (47.3)	48 (18.3)	
16–64	4925 (46.9)	122 (45.3)	
65–74	198 (1.9)	21 (7.6)	
≥ 75	412 (3.9)	83 (28.9)	
Male sex, n (%)	5514 (52.5)	144 (54.8)	0.47
Comorbidities, n (%)			
Hypertension	219 (2.1)	22 (8.4)	< 0.001
Diabetes mellitus	85 (0.8)	15 (5.7)	< 0.001
Hyperlipidemia	64 (0.6)	5 (1.9)	0.03
Gout	19 (0.2)	3 (1.1)	0.02
Chronic lung disease	395 (3.8)	9 (3.4)	0.77
Myocardial infarction	14 (0.1)	2 (0.8)	0.06
Heart failure	13 (0.1)	3 (1.1)	< 0.001
Liver disease	23 (0.2)	2 (0.8)	0.12
Cerebral infarction	49 (0.5)	13 (4.9)	< 0.001
Cancer	141 (1.3)	16 (6.1)	< 0.001
Dementia	28 (0.3)	10 (3.8)	< 0.001

*IQR* interquartile range

The proportions of patients aged 65–74 and ≥ 75 years were higher in the undertriaged group than in the appropriately triaged group. The proportion of patients with hypertension, diabetes mellitus, hyperlipidemia, gout, heart failure, cerebral infarction, cancer, and dementia was higher in the undertriaged group than in the appropriately triaged group.

### Associated factors for undertriage

The results of the multivariable logistic regression analyses are shown in Table 2. Patients aged 16–64, 65–74, and ≥ 75 years (adjusted odds ratio {OR}: 2.40 [95% confidence interval {CI} 1.71–3.36], 8.57 [95% CI 4.83–15.2], and 14.9 [95% CI 9.65–23.01], respectively; reference patients aged < 15 years) and those with diabetes mellitus (adjusted OR, 2.31 [95% CI 1.25–4.26]), dementia (adjusted OR 2.32, [95% CI 1.05–5.10]), and a history of cerebral infarction (adjusted OR 1.98, [95% CI 1.01–3.87]) were more likely to be undertriaged. The results of the sensitivity analyses performed to exclude patients aged 0–15 years showed similar results.

**Table 2:** Multivariable regression analysis of factors associated with undertriage

Variable	Adjusted OR	95% CI	*p* value
Age category, n (%)			
0–15	1 (reference)		
16–64	2.40	1.71–3.36	< 0.001
65–74	8.57	4.83–15.2	< 0.001
≥ 75	14.9	9.65–23.01	< 0.001
Sex			
Male	1 (reference)		
Female	0.90	0.70–1.17	0.43
Comorbidities			
Hypertension	0.94	0.56–1.59	0.82
Diabetes mellitus	2.31	1.25–4.26	0.008
Hyperlipidemia	0.87	0.32–2.35	0.78
Gout	3.55	0.97–13.0	0.056
Chronic lung disease	1.02	0.51–2.03	0.96
Myocardial infarction	1.32	0.28–6.35	0.73
Heart failure	1.14	0.30–4.28	0.85
Liver disease	1.81	0.40–8.26	0.45
Cerebral infarction	1.98	1.01–3.87	0.046
Cancer	1.20	0.67–2.15	0.54
Dementia	2.32	1.05–5.10	0.04

*CI* confidence interval; *OR* odds ratio

## Discussion

In this study, we found that 1) undertriage occurred in 1.3% of patients who were classified by the telephone triage as “hospital visit required” and 2) patients with old age, diabetes mellitus, dementia, and a history of cerebral infarction were at risk of undertriage.

Securing a safe and efficient telephone triage is challenging as a balance between an acceptable low level of overtriage and a minimal level of undertriage needs to be maintained for high patient safety [8]. Thus, investigating risk factors for undertriage in telephone triage is essential for patient safety.

### Proportion of Undertriage

We found that the proportion of undertriage was < 2%. A previous systematic review showed that telephone undertriage occurred in 10% of AHHC medical service cases [9]. Meanwhile, recently, an AHHC service in Copenhagen reported that the incidence of undertriage was 0.04% [11]. It is difficult to compare undertriage rates across studies owing to differences in the definition of undertriage and institutional triage protocols [3]; this may be because there are dozens of telephone triage protocols, and the telephone triage protocol utilized varies across countries or communities [3]. In addition, modified telephone triage protocols have been developed [17, 18], leading to a decrease in undertriage. Thus, telephone triage protocols adapted in each country, area, or modified telephone triage should be evaluated with regard to the level of triage and according to each telephone triage protocol.

### Risk factors associated with Undertriage

We found that old age, diabetes mellitus, dementia, and a history of cerebral infarction were associated with undertriage. A previous study conducted at a trauma center showed that age ≥ 65 years [19], female sex, dementia [20], history of cerebral infarction, and heart failure [21] were risk factors for undertriage of trauma patients. Among these risk factors for undertriage of trauma patients, old age, dementia, and history of stroke were also identified as risk factors in our study.

The telephone triage protocols are used to carefully record and document the patient’s medical history, including hypertension, diabetes mellitus, myocardial infarction, heart failure, liver disease, chronic lung disease, and collagen disease, as well as age ≥ 65 years and the AHHC medical service used in the telephone protocols. However, age ≥ 75 years and history of cerebral infarction are not factors incorporated in the telephone triage protocol. In Japan, the population is rapidly aging, leading to an increased incidence of dementia and cerebral infarction [22]; thus, the patient may not be able to report their symptoms well or a caregiver may not be able to make the call or report their patients’ symptoms well. Therefore, adding dementia or history of cerebral infarction to the telephone protocol may improve the accuracy of telephone triage.

### Limitations

This study has several limitations. First, the study included a relatively small number of undertriaged patients; thus, we could not stratify and analyze each telephone triage level and protocol (Supplementary File 2), including decision-making factors. Second, this study was performed in a single AHHC medical service in Japan; thus, it may be difficult to adapt our results to other triage levels and other countries. However, our approach would be useful to other telephone triage systems to evaluate factors associated with undertriage in other countries. Third, this was a retrospective cohort study; therefore, missing or residual confounding factors could have caused undertriage. However, we believe that important confounding factors (e.g., age ≥ 65 years, heart failure, history of stroke, and dementia), which have been previously reported to cause undertriage, were covered in the present study. Fourth, we did not evaluate inter-rater reliability among AHHC medical service doctors. Fifth, the AHHC medical service could not conduct a follow-up of patients assessed in the undertriaged group sent to the hospital. If very few patients sent to the hospital are admitted and most are sent home, then the telephone triage protocols may well be working and some physicians may in fact be overtriaging. Lastly, the level of triage may have changed by the time the AHHC doctor arrived; the reason for this change should be investigated in the future.

## Conclusion

Telephone triage in an AHHC should be carefully performed for patients with advanced age, dementia, and a history of cerebral infarction. Further studies are warranted to evaluate other telephone triage protocols.

## Supplementary Information

**Additional file 1: File 1.** All patients classified as grades 1, 2, and 3

**Additional file 2: File 2.** Protocol causing undertriage

## Data Availability

The datasets used and/or analyzed during the current study are available from the corresponding author on reasonable request.

## Abbreviations

AHHC: After-hours house call
CI: confidence interval
IQR: interquartile range
OR: odds ratio
