# Supplementary material for: Factors associated with undertriage in patients classified by the need to visit a hospital by telephone triage: a retrospective cohort study
Source: BMC Emerg Med. 2021 Dec 15;21:155. doi: 10.1186/s12873-021-00552-x (PMC8672574; doi:10.1186/s12873-021-00552-x)
Supplement: Supplementary file 1 — Additional file 1: File 1. All patients classified as grades 1, 2, and 3 [file 12873_2021_552_MOESM1_ESM.docx]

Supplementary File 2

Grade 1, 2, and 3 patients

|  | Grade 1 N=9,017 | Grade 2  N=10,479 | Grade 3 N=263 |
| --- | --- | --- | --- |
| Age, years |  |  |  |
| Median (IQR) | 10 (3–33) | 21 (4–37) | 46 (26–78) |
| Category, n (%) |  |  |  |
| 0–15 | 4,906 (54.3) | 4,960 (47.3) | 48 (18.3) |
| 16–64 | 3,707 (41.1) | 4,925 (46.9) | 122 (45.3) |
| 65–74 | 167 (1.9) | 198 (1.9) | 21 (7.6) |
| >75 | 251 (2.8) | 412 (3.9) | 83 (28.9) |
| Male sex, n (%) | 4,689 (52.0) | 5,507 (52.6) | 144 (54.8) |
| Comorbidities, n (%) |  |  |  |
| Hypertension | 145 (1.6) | 219 (2.1) | 22 (8.4) |
| Diabetes mellitus | 65 (0.7) | 85 (0.8) | 15 (5.7) |
| Hyperlipidemia | 51 (0.6) | 64 (0.6) | 5 (1.9) |
| Gout | 14 (0.2) | 19 (0.2) | 3 (1.1) |
| Chronic lung disease | 307 (3.4) | 395 (3.8) | 9 (3.4) |
| Myocardial infarction | 10 (0.1) | 14 (0.1) | 2 (0.8) |
| Heart failure | 3 (0.0) | 13 (0.1) | 3 (1.1) |
| Liver disease | 19 (0.2) | 23 (0.2) | 2 (0.8) |
| Cerebral infarction | 26 (0.3) | 49 (0.5) | 13 (4.9) |
| Cancer | 114 (1.3) | 141 (1.4) | 16 (6.1) |
| Dementia | 24 (0.3) | 28 (0.3) | 10 (3.8) |
| Time from patients’ phone call to doctors’ consultation, min |  |  |  |
| median (IQR) | 115 (57–205) | 112 (55–205) | 81 (40–159) |
