# Supplementary material for: Factors associated with undertriage in patients classified by the need to visit a hospital by telephone triage: a retrospective cohort study
Source: BMC Emerg Med. 2021 Dec 15;21:155. doi: 10.1186/s12873-021-00552-x (PMC8672574; doi:10.1186/s12873-021-00552-x)
Supplement: Supplementary file 2 — Additional file 2: File 2. Protocol causing undertriage [file 12873_2021_552_MOESM2_ESM.docx]

Supplementary File 2.

| Adult (>15 years) | |  | Child (0–15 years) | |
| --- | --- | --- | --- | --- |
| Chief complaint | N=215 |  | Chief complaint | N=48 |
| Common cold symptoms | 45 |  | Fever | 16 |
| Sore throat | 33 |  | Cough | 8 |
| Syncope | 19 |  | Abdominal pain | 5 |
| Diarrhea | 15 |  | Vomiting or nausea | 3 |
| Allergy | 11 |  | Upper extremity problem | 2 |
| Bruising | 11 |  | Lower extremity problem | 2 |
| Tinnitus | 8 |  | Bruising | 2 |
| Lower extremity problem | 7 |  | Rash | 2 |
| Headache | 6 |  | Common cold symptoms | 1 |
| Constipation | 5 |  | Hyperventilation | 1 |
| Foot problem | 5 |  | Laceration | 1 |
| Fever | 4 |  | Fall | 1 |
| Upper extremity problem | 4 |  | Trauma on face or extremities | 1 |
| Dyspnea | 3 |  | Asthma attack symptoms | 1 |
| Rash | 3 |  | Dyspnea | 1 |
| Hematemesis, melena, or blood in stool | 3 |  | Headache | 1 |
| Dizziness | 3 |  |  |  |
| Heat stroke | 3 |  |  |  |
| Wheezing | 2 |  |  |  |
| Abdominal pain | 2 |  |  |  |
| Vomiting or nausea | 2 |  |  |  |
| Urological diseases | 2 |  |  |  |
| Hyperventilation | 2 |  |  |  |
| Laceration | 2 |  |  |  |
| Fall | 2 |  |  |  |
| Disturbance of consciousness | 1 |  |  |  |
| Chest pain | 1 |  |  |  |
| Back pain | 1 |  |  |  |
| Dysarthria | 1 |  |  |  |
| Heartburn | 1 |  |  |  |
| Abnormally colored Urine | 1 |  |  |  |
| Itching | 1 |  |  |  |
| Anxiety or fearfulness | 1 |  |  |  |
| Nasal bleeding | 1 |  |  |  |
| Bleeding | 1 |  |  |  |
| Burn | 1 |  |  |  |
| Wound healing and infection | 1 |  |  |  |
| Trauma on face or extremities | 1 |  |  |  |
